# Canine leishmaniasis in the Atlantic Rainforest Biome region of Bahia, Brazil, affected by deforestation: a one health perspective

**DOI:** 10.1590/S1984-29612025077

**Published:** 2026-02-27

**Authors:** Everton Rusciolelli Nascimento, José Bryan Rihs, Andressa Mariana Saldanha Elias, Marcelo Eduardo Cardozo, João Gabriel Acioli Siqueira, Luísa Mourão Dias Magalhães, Luanna Chácara Pires, Gisele Lopes de Oliveira, Lilian Lacerda Bueno, Ricardo Toshio Fujiwara, Ana Laura Grossi de Oliveira, Sebastião Rodrigo Ferreira

**Affiliations:** 1 Universidade Federal do Sul da Bahia, Centro de Formação em Ciências da Saúde, Teixeira de Freitas, BA, Brasil; 2 Universidade Federal de Minas Gerais, Instituto de Ciências Biológicas, Departamento de Parasitologia, Laboratório de Imunobiologia e Controle de Parasitos, Belo Horizonte, MG, Brasil; 3 Universidade Federal de Minas Gerais, Instituto de Ciências Biológicas, Departamento de Parasitologia, Laboratório de Interações em Imunoparasitologia, Belo Horizonte, MG, Brasil; 4 Universidade Federal do Sul da Bahia, Centro de Formação em Desenvolvimento Territorial, Teixeira de Freitas, BA, Brasil

**Keywords:** Canine visceral leishmaniasis, Leishmania infantum, molecular testing, public health, one health, rKDDR-plus serology, Leishmaniose visceral canina, Leishmania infantum, testes moleculares, saúde pública, saúde única, sorologia rKDDR-plus

## Abstract

Canine visceral leishmaniasis (CVL) is a zoonotic disease whose etiological agent is transmitted by phlebotomine sand flies, with dogs as the primary reservoir. Urbanization and deforestation have created conditions favorable to pathogen transmission. In Eunápolis, Bahia, the epidemiological situation of CVL remains entirely unknown. In this study, the prevalence of CVL and its associated socio-environmental factors were investigated. A cross-sectional study was conducted involving 243 dogs residing in both urban and rural areas. Blood samples were analyzed using the rKDDR-plus immunochromatographic test, conventional polymerase chain reaction (cPCR), and quantitative real time polymerase chain reaction (qPCR). In addition, household questionnaires were administered to assess socio-environmental conditions. Based on the rKDDR-plus serology, four samples tested positive (1.6%), and qPCR confirmed the presence of *Leishmania infantum* DNA in three samples (1.2%). Among the environmental factors analyzed, improper waste management (38.3%) and proximity of households to vegetation (54.7%) were notable. The detection of seropositive samples and *L. infantum* DNA suggests the existence of a transmission cycle of canine visceral leishmaniasis (CVL) in the studied area. In this context, public health measures, including vector control, health education initiatives, and responsible pet ownership, are essential to mitigate risks and prevent the spread of the disease.

## Resumo

A leishmaniose visceral canina (LVC) é uma doença zoonótica cujo agente etiológico é transmitido por flebotomíneos, tendo o cão como principal reservatório. A urbanização e o desmatamento criaram condições favoráveis à transmissão do patógeno. Em Eunápolis, Bahia, a situação epidemiológica da LVC permanece totalmente desconhecida. Neste estudo foi pesquisada a prevalência da LVC e seus fatores socioambientais associados. Foi realizado um estudo transversal com 243 cães residentes em áreas urbanas e rurais. Amostras de sangue foram analisadas por: teste de imunocromatografia rKDDR-plus, reação em cadeia da polimerase convencional (cPCR), reação em cadeia da polimerase quantitativa em tempo real (qPCR). Além disso, foram aplicados questionários domiciliares às condições socioambientais. Através da sorologia rKDDR-plus foram identificadas quatro amostras positivas (1,6%), e a qPCR confirmou DNA de *Leishmania infantum*. em três amostras (1,2%). Entre os fatores ambientais analisados, destacaram-se o manejo inadequado de resíduos (38,3%) e a proximidade das residências à vegetação (54,7%). A detecção de amostras soropositivas e DNA de *L infantum* sugere a existência de um ciclo de transmissão da LVC na área estudada. Nesse contexto, medidas de saúde pública, incluindo o controle vetorial, ações de educação em saúde e a posse responsável de animais de estimação, são fundamentais para reduzir os riscos e prevenir a disseminação da doença.

## Introduction

Leishmaniasis, a parasitic disease of zoonotic origin, is caused by protozoa of the genus *Leishmania.* These protozoa are transmitted to hosts through the bite of infected sand flies (Gontijo & Carvalho 2003), which thrive in moist soil or decomposing organic matter under low-light conditions. The sand flies are most active during crepuscular hours, typically at dusk and dawn. Canine visceral leishmaniasis, caused by *Leishmania infantum,* represents an important public health concern due to its role in the epidemiological cycle of human leishmaniasis. Domestic dogs, acting as the primary urban reservoir, can exhibit a wide range of clinical manifestations, from subclinical infections to severe systemic involvement. The high level of cutaneous parasitic load in infected canines facilitates the transmission of the parasite to sand flies, thereby perpetuating the cycle of transmission to humans (Soares et al., 2022; Vilas-Boas et al., 2024).

One Health is established on the interconnectedness among human, animal, and environmental health, advocating for collaborative and multidisciplinary approaches to address complex health challenges (Parodi 2021; Azhar 2023). Diseases such as leishmaniasis illustrate the imperative for this approach, as their development is influenced by interactions among these domains (Gibbs 2014; Mackenzie & Jeggo 2019). Environmental changes such as deforestation, urbanization, and inadequate sanitation have contributed to the proliferation of sand flies, thereby shifting the epidemiological pattern of leishmaniasis from rural to urban areas. These transformations, in combination with migratory movements and the adaptation of sand flies to human environments, emphasize the importance of integrated strategies to control the disease (Moreira et al., 2003; Barbosa et al., 2016).

The diagnosis of CVL requires a combination of clinical evaluation and laboratory methods. Serological tests are commonly employed for screening due to their simplicity, cost-effectiveness, and rapid turnaround time. However, these tests present some limitations, including low sensitivity in subclinical or early-stage cases and the potential for cross-reactivity (Carvalho et al., 2018; Gondim et al., 2022). Molecular techniques, such as polymerase chain reaction (PCR), are employed for confirmation, offering higher sensitivity and specificity by detecting the parasite’s genetic material in animal tissues (Gondim et al., 2022).

The complexity of urban transmission has hindered the effectiveness of control measures, such as the culling of infected animals, in interrupting disease transmission. The factors driving urban transmission chains are reportedly more intricate and multifaceted than those observed in rural settings (Sousa et al., 2018). To address this challenge, it is essential to implement comprehensive strategies that include effective vector and transmission control measures. Among the vector control actions, regular cleaning of backyards, vacant lots, and public squares stands out, involving the removal of leaves, branches, and other debris that may serve as shelter for sand flies, as well as the proper disposal of waste and the prevention of organic matter accumulation in the soil, thereby reducing vector breeding sites. In terms of transmission control, the use of insecticide-impregnated collars on dogs is recommended to lower parasite loads and reduce the likelihood of parasite transmission to the vector. Individual protection measures are also important, such as installing screens on doors and windows, using insecticide-treated mosquito nets and topical repellents, especially during periods of peak sand fly activity, in addition to early diagnosis and treatment to interrupt the transmission cycle (Brasil, 2024).

The study conducted by Ribeiro et al. (2021) revealed that visceral leishmaniasis remains a persistent public health problem in the Northeast, with an upward trend in the last decade. Its geographical distribution is heterogeneous, with clusters of high incidences forming in socially vulnerable areas. Eunápolis, located in the state of Bahia, Brazil, characterized by unfavorable socioeconomic conditions and a lack of awareness about the epidemiological situation of the CVL, urgently requires studies to verify the occurrence of visceral leishmaniasis. This is essential for implementing effective prevention and control policies.

Current control efforts have proven inadequate in preventing the spread of CVL, emphasizing the need for a deeper understanding of its epidemiological dynamics in urban environments such as the Eunápolis city region (Oliveira et al., 2021). The role of domestic dogs in maintaining the transmission cycle is further complicated by their increasing presence in households and the lack of public policies addressing stray and domestic animal populations. These challenges highlight the complexity of managing leishmaniasis in urbanized settings. This study aimed to estimate the prevalence of L. infantum-infected dogs in Eunápolis and to provide preliminary evidence of local transmission. The findings are expected to improve understanding of the drivers of CVL transmission in the region and to inform control strategies.

## Materials and Methods

### Study site and sample composition

A cross-sectional study was conducted between October and December 2023 in Eunápolis, Bahia, within Brazil's Deforestation-affected Atlantic Rainforest Biome. The municipality encompasses an area of 1.425,970 km^2^, geographical coordinates: Latitude: -16.3731, Longitude: -39.5751 16° 22 23 S, 39° 34 30 W (IBGE, 2022). The study population consisted of 243 dogs from Eunápolis, comprising both sexes, aged one year or older, and various breeds. The dogs either presented with or were free from clinical signs of CVL, and had no history of blood transfusions or travel to endemic areas. All dogs were under the care of responsible owners; however, some animals spent part of the day outside the household premises, having free access to the peridomestic or surrounding areas. Given that this is a pioneering study based on non-probabilistic convenience sampling, the sample size (n) was determined using the OpenEpi® software, applying the following equation, where N represents the population size (accounting for finite population correction), p denotes the hypothetical percentage frequency of the event in the population, EDFF refers to the design effect for cluster surveys, and d indicates the margin of error (confidence limit) expressed as an absolute percentage: 


$$n\;=\;\left[EDFF\;\times \;N\;\times \;p\;\times \;\left(1\;-\;p\right)\right]\;/\;[(d²\;/\;Z²(1-\alpha /2)\;\times \;\left(N\;-\;1\right))\;+\;p\;\times \;\left(1\;-\;p\right)]$$ (1)

The project was approved by the Animal Use Ethics Committee of the Federal University of Minas Gerais (UFMG) under protocol number 330/2022.

### Clinical evaluation and sample collection

Clinical evaluations were conducted at the dogs’ households to identify potential clinical signs associated with CVL, including skin lesions, onychogryphosis, lymphadenomegaly, ocular alterations, reduced body condition scores, and elevated body temperature. Following the assessments, blood samples were collected, numbered from 1 to 243, and stored in tubes containing EDTA anticoagulant, with a volume of 1 mL per kilogram of body weight, capped at a maximum of 5 mL. The animals were identified with sequential numbering according to the order of collection, and the corresponding address was recorded in written form, together with the geographical coordinates obtained using a mobile phone through the Google Maps application.

### rKDDR-plus immunochromatographic assay

The rKDDR-plus immunochromatographic assay (Safetest® Diagnósticos, Brazil) was performed according to the manufacturer's guidelines (Siqueira et al., 2021). In summary, 20 µL of serum was applied to the test device, followed by the addition of one drop of buffer solution. Results were interpreted after a 10-minute incubation period. The appearance of two red lines indicated a positive result, whereas a single red line signified a negative result.

### DNA extraction and PCR

DNA extraction from the 243 samples was performed according to the Trizol™ protocol (Thermo Fisher Scientific, USA). The samples consisted of small volumes of frozen blood, and the use of Trizol™ protocol allowed simultaneous isolation of DNA and RNA with minimal loss of genetic material, ensuring the efficient and rational use of the available material. Although Trizol™ is traditionally employed for RNA extraction, it is also possible to recover DNA from the interphase and organic phase, provided that additional purification and hydration steps are applied, as originally described by Chomczynski (1993).The conventional polymerase chain reaction (cPCR) was performed using 4 μL of Promega™ GoTaq 5x (5 U/μL), 2μL Invitrogen™ dNTPs (10mM), 1μL primers (10μM) of 150 (5'-GG)G (G/T) AG GGG CGT TCT (G/C)CG AA-3') and 152 (5'-(G/C)(G/C)(G/C)(A/T)CT AT(A/T) TTA CAC CAA CCC C-3'), 0.25μL Invitrogen™ (5U/μL) and water RNA free (up to 10μL), which were designed to amplify kDNA minicircles of *Leishmania* (200ng/uL), resulting in an amplicon of approximately 120 base pairs. *L. infantum* DNA (200ng/uL) was used as an infected control. The cycling conditions included an initial denaturation at 95°C for 5 minutes, followed by 29 cycles of denaturation at 95°C for 1 minute, annealing at 55°C for 1 minute, and extension at 72°C for 1 minute, with a final extension at 72°C for 5 minutes. DNA fragment amplification was assessed by agarose gel electrophoresis (1.5%), stained with ethidium bromide, and visualized under ultraviolet light. Specific amplification bands were verified using a 100-base-pair (bp) molecular weight marker (Biorad™).

### Real-time PCR (qPCR) analysis

Positive samples in the immunochromatographic assay were subjected to quantitative polymerase chain reaction (qPCR). Reactions were carried out in 96-well plates on a 7500 Real-Time PCR thermocycler (Applied Biosystems), using 1 µL of sample (100 ng), 5 µL of SYBR™ Green PCR Mastermix (Applied Biosystems), 0.3 µL of each primer sequence of cPCR (10 µM), and ultrapure water to complete the final volume of 10 µL. The primers used were the same as those employed in cPCR. The qPCR cycling program followed the standard conditions: initial denaturation at 95°C for 10 minutes, followed by 40 cycles of denaturation at 95°C for 15 seconds, and extension at 60°C for 1 minute. After the reaction, melting curves were generated and analyzed. Positive and negative controls, using *L. infantum* DNA, were included for comparison. The quantification of the samples was determined using the standard curve method for absolute quantification. The standard curves were prepared using a serial dilution of DNA extracted from 106 promastigotes of *L. infantum*. The results were expressed as the number of parasites per milliliter (mL) of extracted tissue DNA.

### Identification of socioenvironmental factors

During the visits to the owners, a questionnaire available at Supplementary Information (S1) was applied to assess the socioenvironmental, socioeconomic, and behavioral conditions that could predispose the dogs to infection by *L. infantum*. The animal guardians were interviewed to gather information about the dog’s origin (local or imported) and medical history, including any previous blood transfusions. The guardians were also asked about their knowledge of the “sand fly”, including its presence in the area, and whether they took preventive measures, such as using mosquito nets. Additionally, the researchers observed and inquired about the presence of other animals in the yard and the proximity of waste disposal sites.

### Data analysis

The seroprevalence (SP) was calculated using the formula: SP = (number of positive dogs/sample size) ×100. Descriptive statistical analyses were conducted to summarize the dataset. The chi-square test or Fisher's exact test was applied to evaluate differences in the occurrence of *L. infantum* infection according to dog and household characteristics. A significance level of 5% (p < 0.05) was adopted. All statistical analyses were performed using R software, with the Stats and Epitools packages. 

## Results and Discussion

**Figure 1:**
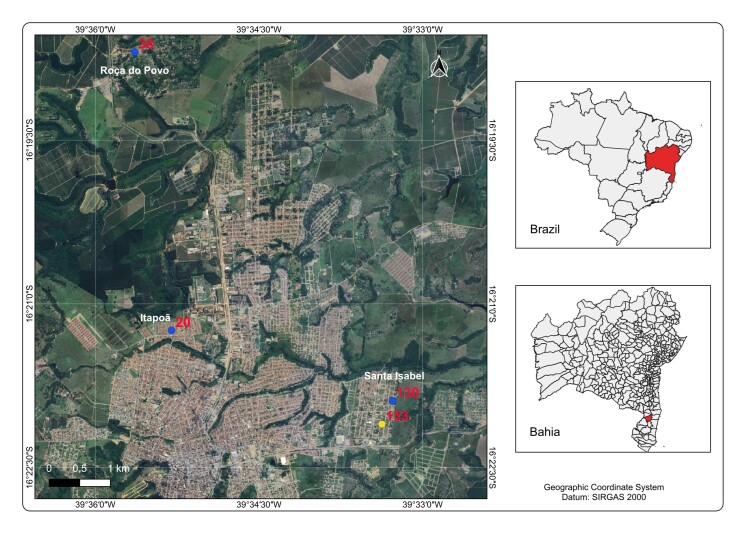
Spatial distribution of canine visceral leishmaniasis (CVL) in Eunápolis, Bahia, Brazil (October–December 2023). Blue points represent dogs positive by both immunochromatographic and molecular assays (qPCR), whereas the single yellow point indicates a dog positive only by immunochromatographic testing. The maps on the right indicate the geographical location of the municipality within Brazil and the state of Bahia.

This study offers novel insights into the serological, molecular, and epidemiological characteristics of CVL in Eunápolis, Bahia. The geographical location of Eunápolis in the Northeastern region of Brazil is presented in Figure 1.

Among the 243 evaluated dogs, 135 (55.6%) were female and 108 (44.4%) were male. The median age of the sampled dogs was 4.0 years [interquartile range (IQR): 2.00–6.7 years], indicating a relatively young study population. Among positive cases, the median age was 2.5 years (IQR 1.6–3.7), suggesting a concentration of infections in younger animals. A chi-square test revealed a statistically significant association between age group and *L. infantum* positivity (p < 0.0001), with 75% of positive cases occurring in dogs ≤3 years old (Table 1).

**Table 1:** Main characteristics of dogs evaluated for Leishmania infantum infection in Eunápolis, Bahia State, Brazil, from October to December 2023.

**Variable**	**Category**	**n**	**%**	**p-value** ^*^	**Positives n (%)**
Sex	Female	135	55.6	0.0833	2 (1.5)
	Male	108	44.4		2 (0.9)
Age-years	0-1	41	16.9	<0.0001	1 (2.4)
	1.1 – 2	26	10.7		1 (3.8)
	2.1 – 3	40	16.4		1 (2.5)
	3.1 – 7.9	97	39.9		1 (1.0)
	>7.9	39	16.0		0 (0.0)
Breed	Mixed breed	190	78.2	<0.0001	3 (1.6)
	Pure breed	53	21.8		1 (1.9)

n: absolute frequency; %: relative frequency; Positives n (%): number (and percentage) of positive dogs within each variable category; *p-value*: Chi-square goodness-of-fit test.

* Chi-square adherence test. Note: The low number of positive cases limits statistical inference. Median age: 4.00 [2.00–6.75] years; Median age among positives: 2.50 [1.625–3.75] years.

Regarding breed, most of the studied animals (78.2%) were mixed-breed dogs, while 21.8% belonged to defined breeds (Table 1). Toscano et al. (2013) observed that *Lutzomyia longipalpis*, a species complex of sand flies in the family Psychodidae, feeds opportunistically, suggesting that infection risk is independent of sex, breed, or age.

**Supplementary Figure 1:**
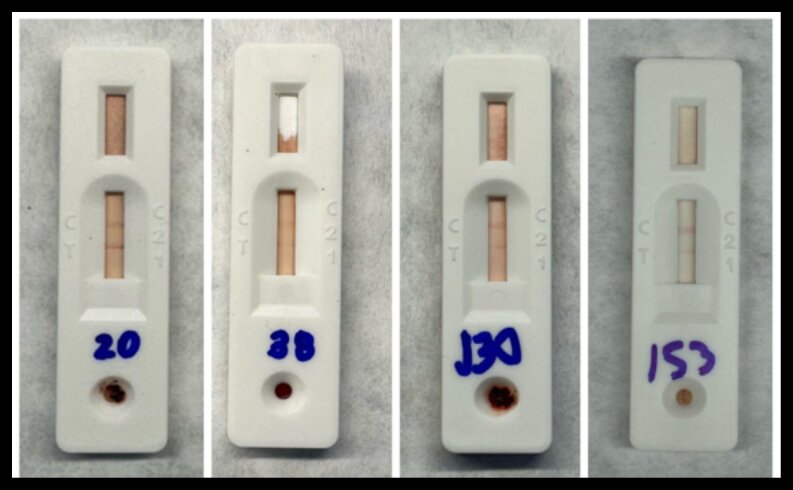
Immunochromatographic test results for anti-rKDDR-plus IgG in samples from dogs that tested positive for Leishmania spp., Eunápolis, Bahia, Brazil (October–December 2023)

**Supplementary Figure 2:**
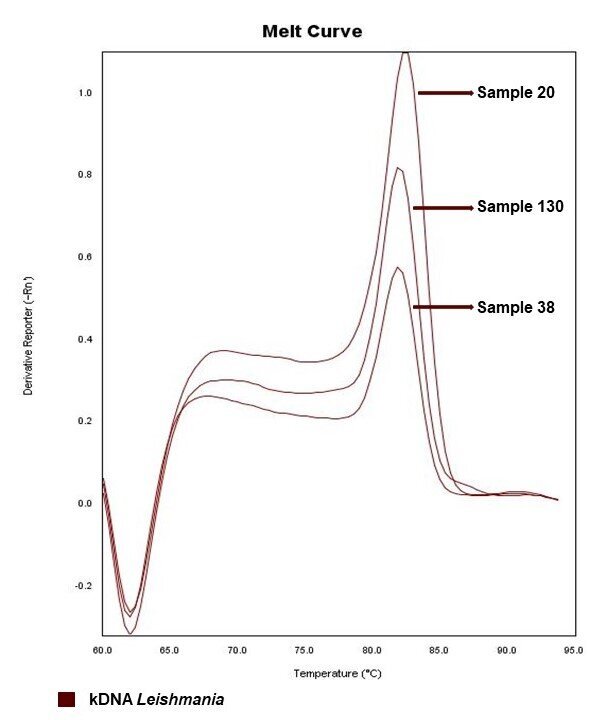
qPCR melting curve analysis showing amplification (Tm) peaks for samples 38 and 130 from canine blood collected in Eunápolis, Bahia, Brazil, from october-december of 2023.

Anti-rKDDR-plus-IgG seropositivity was observed in four dog samples (IDs 20, 38, 130, and 153), corresponding to a seroprevalence of 1.6% (Supplementary Figure 1). All 243 samples were tested by cPCR, and those that tested positive in the immunochromatographic assay were additionally tested by qPCR. The cPCR did not detect *L. infantum* DNA in any specimen, whereas qPCR detected *L. infantum* DNA in three samples (IDs 20, 38, and 130), with parasite loads of 84.7, 71.9, and 80.6 parasites/mL, respectively, indicating low-level parasitemia. Melting-curve analysis (Supplementary Figure 2) confirmed specific amplicon peaks, underscoring the higher analytical sensitivity of qPCR to detect low parasite burdens compared with cPCR, particularly in subclinical infections. The use of blood samples for molecular diagnostics offers practical advantages, including ease of collection and reduced invasiveness. However, this approach may have underrepresented the true prevalence of the disease, as the parasite burden in circulating blood tends to be low during certain periods after infection (Tavares et al., 2011). Overall, the combined prevalence of serological and molecular positivity was 1.2%. This prevalence suggests that Eunápolis is in the initial stages of local CVL transmission, categorizing it as a silent yet vulnerable and receptive region for further cases, as classified by Moreira et al. (2003).

The collection sites of dogs that tested positive using immunochromatographic and qPCR assays were mapped (Figure 1). The collection point for Dog 38, located furthest away, was situated in the rural area known as Roça do Povo. Point 20 was located in the Itapoã neighborhood, while points 130 and 158 were in a peripheral area of the city called Santa Isabel, at the home of an animal hoarder. All these areas were close to forested zones and had a high canine population density.

The clinical characteristics observed during the evaluation of the dogs in this study are presented in Table 2, highlighting the distribution of clinical signs, such as nutritional status, skin lesions, and other relevant manifestations. Among the clinically evaluated animals, 235 (95.9%) showed no lesions characteristic of CVL. Of the eight (3.3%) dogs with skin manifestations suggestive of CVL, six (2.5%) presented diffuse lesions across the body, while one (0.4%) had lesions on the muzzle and another (0.4%) on the back. Of the 62 (25.4%) dogs displaying clinical signs compatible with CVL, the most common were malnutrition/weight loss in 26 (10.6%), onychogryphosis in eight (3.3%), and abdominal distension in another eight (3.3%). Less frequent signs included lymphadenopathy in the popliteal lymph node in five (2.0%), anorexia in four (1.6%), and conjunctivitis in one dog (0.4%).

**Table 2:** Clinical parameters of dogs evaluated for *Leishmania infantum* infection in Eunápolis, Bahia State, Brazil, from October to December 2023.

**Clinical parameter**	**Category**	**n**	**%**	**p-value** ^*^	**Positives n (%)**
Nutritional *status*	Optimal	55	22.6	<0.0001	0 (0.0)
	Good	110	45.3		2 (1.8)
	Regular	61	25.1		2 (3.3)
	Poor	17	7.0		0 (0.00)
Presence of lesions	No	235	95.9	<0.0001	3 (1.2)
	Yes	8	3.3		1 (12.5)
	Diffuse	6	2.5		0 (0.0)
	Muzzle	1	0.4		1 (100.0)
	Back	1	0.4		0 (0.0)
Clinical sign	Alopecia	10	4.1	<0.0001	0 (0.0)
	Onychogryphosis	8	3.3		1 (12.5)
	Anorexia	4	1.6		0 (0.0)
	Abdominal Distention	8	3.3		0 (0.0)
	Conjunctivitis	1	0.4		0 (0.0)
	Weight Loss	26	10.6		2 (7.7)
	Lymphadenopathy	5	2.0		1 (20.0)

n: absolute frequency; %: relative frequency; Positives n (%): number (and percentage) of positive dogs within each variable category; *p-value*: Chi-square goodness-of-fit test.

* Chi-square adherence test. Note: The low number of positive cases limits statistical inference.

However, the four seroreactive dogs identified in this study exhibited only mild clinical signs of CVL, likely due to their young age and low parasite burden. Although the clinical manifestations of CVL often follow typical patterns, many signs overlap with those of other infectious diseases, metabolic disorders, and dermatological conditions (Fernandez et al., 2008; Ziener et al., 2008).

The chi-square goodness-of-fit test revealed significant results for optimal, good, and normal nutritional conditions, as well as for the absence of lesions, while poor nutritional status and the presence of lesions were less frequently observed and not statistically significant. This likely reflected the domiciled and well-cared-for nature of the animals included in this study. Regarding the statistical difference between the observed frequencies and the expected proportion among positive animals, poor or regular nutritional condition was anticipated. However, the findings revealed that the animals had either good or normal nutritional status. This discrepancy may be attributed to the young age of the animals, suggesting a recent or non-latent infection. Consequently, these animals exhibited no skin lesions and only subtle clinical signs, with weight loss emerging as the most prominent sign of CVL (Meléndez-Lazo et al., 2018).

Descriptive data on socio-environmental factors related to the presence of *Lu. longipalpis*, the vector of CVL, are presented in Table 3, and an aerial view of the municipality of Eunápolis showing urban and rural sites is provided in Figure 1. Regarding animal breeding near households, 126 (51.8%) dog owners reported no nearby animal rearing, while 117 (48.1%) reported its presence, including poultry (chickens, ducks, and birds) and, in some rural areas, cattle, horses, and pigs. The presence of domestic animals, particularly poultry, has been linked to increased peridomestic vector activity (Belo et al., 2013). Although these animals are not proven reservoirs, they significantly contribute to maintaining the *Lu. longipalpis* life cycle by promoting organic matter accumulation and serving as sources of blood meals (Dias et al., 2003; Araujo et al., 2016). Regarding the surrounding environment, 133 (54.7%) of the residences were located near secondary vegetation and 110 (45.3%) close to primary vegetation. In addition, 93 (38.3%) households had some form of waste accumulation in their yards, and 173 (71.2%) were adjacent to vacant lots or construction sites containing dense vegetation, debris, or household refuse. These environmental conditions, characterized by deforestation, urbanization, and unplanned settlement, favor the vector proliferation and increase the risk of phlebotomine sand fly presence near human dwellings (Araujo et al., 2016; Santos et al., 2021).

**Table 3:** Socioenvironmental factors associated with canine leishmaniasis in Eunápolis, Bahia State, Brazil, from October to December 2023.

**Variable**	**Category**	**n**	**%**	**p-value** ^*^	**Positives n (%)**
Animal husbandry close to home	No	126	51.8	1.0000	1 (0.8)
	Yes	117	48.1		3 (2.5)
Presence of vegetation in the area	Secondary Vegetation	133	54.7	0.5493	2 (1.5)
	Primary Vegetation	110	45.3	1.0000	2 (1,8)
Presence of waste in the area	No	150	61.7	0.7193	1 (0.7)
	Yes	93	38.3		3 (3.2)
Vacant house lots in the area	No	70	28.8	1.000	0 (0.0)
	Yes	173	71.2		4 (2.3)
Insect and mosquito protection net	No	228	93.8	0.3219	3 (1.3)
	Yes	15	6.2		1 (6.7)
Knowledge about CVL	No	182	74.9	0.195	1 (0.5)
	Yes	61	25.1		3 (4.9)

n: absolute frequency; %: relative frequency; Positives n (%): number (and percentage) of positive dogs within each variable category; *p-value*: Chi-square goodness-of-fit test.

* Chi-square adherence test. *Chi-square adherence test. Note: The low number of positive cases limits statistical inference.

Since the 1940s, Eunápolis has experienced significant changes, primarily driven by the establishment of companies exploiting timber resources and engaging in livestock farming. This urbanization has led to the depletion of native forests, contributing to increased deforestation in the region (Oliveira et al., 2021).

The mosquito nets were present in only 15 (6.2%) households, of which 10 (4.1%) were in rural areas. However, conventional mosquito nets provide limited protection against phlebotomine sand flies, as their typical mesh size allows these smaller insects to pass through. Effective protection is achieved only when the netting is treated with residual pyrethroids (Alexander & Maroli 2003; Abbott et al., 2025). Although the use of insecticide-impregnated collars is an effective measure to prevent infection in dogs and is recommended by the Ministry of Health (Brasil 2024), no dog owner reported using this preventive method. Therefore, this control measure should be more widely disseminated. Preventing sand fly bites remains essential for interrupting *Leishmania* transmission (Miró et al., 2008).

Among the interviewees, 182 (74.9%) had no prior knowledge of leishmaniasis or its vector and were unaware of its transmission methods. This lack of knowledge leads to the failure to adopt appropriate preventive measures and delays healthcare seeking and diagnosis, contributing to the delayed initiation of treatment (Barbosa et al., 2016). Although only a chi-square goodness-of-fit test was applied, comparing the distribution of responses across categories, it is noteworthy that individuals who reported prior knowledge of leishmaniasis had a higher proportion of positive cases (4.9%) compared with those who were unaware (0.5%). This unexpected result may reflect greater exposure to areas with an increased probability of disease occurrence rather than the effect of knowledge itself and thus warrants further investigation.

This study reinforces the need for a One Health approach to control CVL in Eunápolis, Bahia, where environmental, animal, and human health are closely interconnected. The likely presence of *Lu. longipalpis* and other phlebotomine species (Rodgers et al., 2019), together with organic matter accumulation and inadequate waste management, favors the occurrence of the disease. The detection of CVL among young, subclinical dogs indicates the presence of silent reservoirs that may sustain infection and precede human cases. Furthermore, limited community awareness regarding CVL and its transmission pathways intensifies the risk, underscoring the need for integrated public health interventions targeting both human and animal health.

This study has limitations that should be acknowledged when interpreting the findings. The relatively small number of CVL-positive cases may have reduced statistical power, particularly for assessing associations with factors such as age, breed, and clinical manifestations. In addition, the cross-sectional design did not allow causal relationships between socio-environmental factors and CVL prevalence to be firmly established. The absence of confirmation of the sand fly species in the study area may also have restricted a more detailed understanding of vector dynamics and transmission patterns. Finally, the reliance on owner-reported information regarding waste management and animal breeding near households could have introduced recall bias, although such data remain valuable for characterizing local practices. Despite these considerations, this study represents a pioneering effort to address the impacts of deforestation in the Southern Atlantic Forest of Bahia, highlighting how environmental changes affect the health of both animals and humans in the context of CVL.

## Conclusions

In Eunápolis, Bahia, the detection of seropositive dogs and *L. infantum* DNA confirms the circulation of CVL in both urban and rural areas. Although prevalence is low, the socio-environmental conditions favor the maintenance and expansion of transmission. It is recommended to intensify surveillance actions, focal investigations, vector control, and health education activities, in accordance with Ministry of Health guidelines. Additional studies, including entomological surveys and longitudinal monitoring, are essential to better understand the local transmission dynamics and support integrated control strategies.

## Supplementary Material

Supplementary material accompanies this paper.

Supplementary Information (S1)

This material is available as part of the online article from https://doi.org/10.1590/S1984-29612025077.

## References

[B001] Abbott A, Chancey RJ, Roy SL, Centers for Disease Control and Prevention (2025). CDC yellow book: health information for international travel..

[B002] Alexander B, Maroli M (2003). Control of phlebotomine sandflies. Med Vet Entomol.

[B003] Araujo AC, Costa AP, Silva IWG, Matos NNVG, Dantas ACS, Ferreira F (2016). Epidemiological aspects and risk factors for infection by *Leishmania infantum chagasi* in dogs from the municipality of Petrolina, Northeastern Brazil. Vet Parasitol Reg Stud Reports.

[B004] Azhar M (2023). Mapping of WHO internal One Health resources/Cartographie des ressources Une seule santé internes à l’OMS. Wkly Epidemiol Rec.

[B005] Barbosa MN, Guimarães EA, Luz ZMP (2016). Avaliação de estratégia de organização de serviços de saúde para prevenção e controle da leishmaniose visceral. Epidemiol Serv Saude.

[B006] Belo VS, Werneck GL, Barbosa DS, Simões TC, Nascimento BWL, Silva ES (2013). Factors associated with visceral leishmaniasis in the Americas: a systematic review and meta-analysis. PLoS Negl Trop Dis.

[B007] Brasil (2024). Guia de vigilância em saúde: volume 2.

[B008] Carvalho FLN, Riboldi EO, Bello GL, Ramos RR, Barcellos RB, Gehlen M (2018). Canine visceral leishmaniasis diagnosis: a comparative performance of serological and molecular tests in symptomatic and asymptomatic dogs. Epidemiol Infect.

[B009] Chomczynski P (1993). A reagent for the single-step simultaneous isolation of RNA, DNA and proteins from cell and tissue samples. Biotechniques.

[B010] Dias FOP, Lorosa ES, Rebêlo JMM (2003). Fonte alimentar sanguínea e a peridomiciliação de *Lutzomyia longipalpis* (Lutz & Neiva, 1912) (Psychodidae, Phlebotominae). Cad Saude Publica.

[B011] Fernandez NJ, Henderson DW, Spotswood T, Christmas R (2008). Multi-systemic disease in a dog. Can Vet J.

[B012] Gibbs EPJ (2014). The evolution of One Health: a decade of progress and challenges for the future. Vet Rec.

[B013] Gondim CN, Ferreira SA, Vasconcelos BKS, Wouters F, Fujiwara RT, Castro JC (2022). Visceral leishmaniasis in a recent transmission region: 27.4% infectivity rate among seronegative dogs. Parasitology.

[B014] Gontijo B, Carvalho MLR (2003). Leishmaniose tegumentar americana. Rev Soc Bras Med Trop.

[B015] IBGE (2022). Área territorial dos municípios 2022.

[B016] Mackenzie JS, Jeggo M (2019). The One Health approach – why is it so important?. Trop Med Infect Dis.

[B017] Meléndez-Lazo A, Ordeix L, Planellas M, Pastor J, Solano-Gallego L (2018). Clinicopathological findings in sick dogs naturally infected with Leishmania infantum: comparison of five different clinical classification systems. Res Vet Sci.

[B018] Miró G, Cardoso L, Pennisi MG, Oliva G, Baneth G (2008). Canine leishmaniosis: new concepts and insights on an expanding zoonosis. Part two. Trends Parasitol.

[B019] Moreira ED, Souza VMM, Sreenivasan M, Lopes NL, Barreto RB, Carvalho LP (2003). Peridomestic risk factors for canine leishmaniasis in urban dwellings: new findings from a prospective study in Brazil. Am J Trop Med Hyg.

[B020] Oliveira JLM, Neto SPGC, Silva JBL (2021). Avaliação das mudanças no uso e ocupação do solo do município de Eunápolis-BA através da análise da eficiência dos índices espectrais de NDVI, NDBI e built-up. Braz J Dev.

[B021] Parodi AL (2021). Le concept « One Health », une seule santé: réalité et perspectives. Bull Acad Natl Med.

[B022] Ribeiro CJN, Santos AD, Lima SVMA, Silva ER, Ribeiro BVS, Duque AM (2021). Space-time risk cluster of visceral leishmaniasis in Brazilian endemic region with high social vulnerability: an ecological time series study. PLoS Negl Trop Dis.

[B023] Rodgers MSM, Bavia ME, Fonseca EOL, Cova BO, Silva MMN, Carneiro DDMT (2019). Ecological niche models for sand fly species and predicted distribution of *Lutzomyia longipalpis* (Diptera: Psychodidae) and visceral leishmaniasis in Bahia state, Brazil. Environ Monit Assess.

[B024] Santos CVB, Sevá AP, Werneck GL, Struchiner CJ (2021). Does deforestation drive visceral leishmaniasis transmission? A causal analysis. Proc Biol Sci.

[B025] Siqueira WF, Viana AG, Reis Cunha JL, Rosa LM, Bueno LL, Bartholomeu DC (2021). The increased presence of repetitive motifs in the KDDR-plus recombinant protein, a kinesin-derived antigen from *Leishmania infantum*, improves the diagnostic performance of serological tests for human and canine visceral leishmaniasis. PLoS Negl Trop Dis.

[B026] Soares PHA, Silva ES, Penaforte KM, Ribeiro RAN, Melo MOG, Cardoso DT (2022). Responsible companion animal guardianship is associated with canine visceral leishmaniasis: an analytical cross-sectional survey in an urban area of southeastern Brazil. BMC Vet Res.

[B027] Sousa JMDS, Ramalho WM, Melo MA (2018). Demographic and clinical characterization of human visceral leishmaniasis in the State of Pernambuco, Brazil, between 2006 and 2015. Rev Soc Bras Med Trop.

[B028] Tavares R, Staggemeier R, Borges A, Rodrigues M, Castelan L, Vasconcelos J (2011). Molecular techniques for the study and diagnosis of parasite infection. J Venom Anim Toxins Incl Trop Dis.

[B029] Toscano CP, Rossi CN, Ribeiro VM, Laurenti MD, Larsson CE (2013). Caracterização clínica e epidemiológica das leishmanioses em cães no Estado de São Paulo. Braz J Vet Res Anim Sci.

[B030] Vilas-Boas DF, Nakasone EKN, Gonçalves AAM, Lair DF, Oliveira DS, Pereira DFS (2024). Global distribution of canine visceral leishmaniasis and the role of the dog in the epidemiology of the disease. Pathogens.

[B031] Ziener ML, Bettenay SV, Mueller RS (2008). Symmetrical onychomadesis in Norwegian Gordon and English setters. Vet Dermatol.

