# Supplementary material for: Canine leishmaniasis in the Atlantic Rainforest Biome region of Bahia, Brazil, affected by deforestation: a one health perspective
Source: Rev Bras Parasitol Vet. 2026 Feb 27;34(4):e013925. doi: 10.1590/S1984-29612025077 (PMC12974784; doi:10.1590/S1984-29612025077)
Supplement: Supplementary Information (S1) [file rbpv-34-4-e013925-suppl1.pdf]

SERVIÇO PÚBLICO FEDERAL – MINISTÉRIO DA EDUCAÇÃO

Universidade Federal do Sul da Bahia (UFSB) – Campus Paulo Freire

Projeto: Aspectos epidemiológicos da Leishmaniose Visceral no município de Eunápolis/BA

Responsável: Everton Rusciolelli Nascimento

Veterinary Intake Form

1 – IDENTIFICATION

ANIMAL'S NAME: ________________ No.: _____ AGE: _____ SEX: ___ BREED: ________________

GUARDIAN: ____

PHONE: ____

ADDRESS: ____

Animal Identification (additional fields)

Name: ________________

Identification number: ________________

Species: ________________

Breed: ________________

2 – ANIMAL ASSESSMENT

Nutritional status: EXCELLENT ( ) GOOD ( ) FAIR ( ) POOR ( )

Lesions present? _________ Lesion location: __________________________

2.1 – SYMPTOMS

ALOPECIA ( ) ONYCHOGRYPHOSIS ( ) INAPPETENCE ( ) ABDOMINAL DISTENSION ( )

CONJUNCTIVITIS ( ) WEIGHT LOSS ( ) LYMPHADENOPATHY ( ) TEMPERATURE: _____

3 – SAMPLE COLLECTION

BLOOD ( ) LESION SCRAPING ( ) SKIN SCRAPING ( ) BONE MARROW ASPIRATION ( )

4 – ANIMAL PROVENANCE

4.1 – BIRTH CITY: _____________________________________

4.2 – VISITED AN ENDEMIC AREA? WHICH: ___________________

Endereço: Universidade Federal de Minas Gerais - Instituto de Ciências Biológicas, bloco E, 4º andar, sala 173. Av. Pres. Antônio Carlos, 6627 - Pampulha, Belo Horizonte – MG

Universidade Federal do Sul da Bahia, Campus Paulo Freire - Praça Joana Angélica, 250, bairro São José, Teixeira de Freitas – BA. e-mail: ppgsab@ufsb.edu.br

4.3 – HAS RECEIVED A BLOOD TRANSFUSION? (YES) (NO)

5 – VECTOR-RELATED DATA

5.1 – COMPLAINTS ABOUT SAND FLIES? (YES) (NO)

5.1.1 – PERIOD OF HIGHEST COMPLAINT: (MORNING) (AFTERNOON) (NIGHT) (NONE)

5.2 – VEGETATION IN THE SURROUNDINGS: PRIMARY (   )   SECONDARY (   )

5.3 – ANIMAL HUSBANDRY IN THE SURROUNDINGS (YES) (NO). WHICH: ________________

5.4 – HAVE YOU HEARD ABOUT CVL? (YES) (NO)

5.5 – GARBAGE PRESENT IN THE YARD? (YES) (NO)

5.6 – VACANT LOT NEAR THE RESIDENCE? (YES) (NO)

5.7 – USE OF MOSQUITO NETS AND SCREENS? (YES) (NO)

6 – TEST RESULTS

IMMUNOLOGICAL (SEROLOGICAL) TEST FOR CVL: ________________

____

Signature of the person responsible for the information provided

Endereço: Universidade Federal de Minas Gerais - Instituto de Ciências Biológicas, bloco E, 4º andar, sala 173. Av. Pres. Antônio Carlos, 6627 - Pampulha, Belo Horizonte – MG

Universidade Federal do Sul da Bahia, Campus Paulo Freire - Praça Joana Angélica, 250, bairro São José, Teixeira de Freitas – BA. e-mail: ppgsab@ufsb.edu.br

DECLARAÇÃO DE CONSENTIMENTO

I have been duly informed about all procedures in this study (visual inspection, measurement of weight and temperature, and blood collection), as well as the risks and benefits to the animal(s) for whom I am responsible. I was also informed that I may withdraw my animal(s) from the study at any time. By signing this Consent Form, I authorize the participation of my animal(s), identified below, in this project.

This document will be signed in two copies, one of which will remain with me and the other with the researcher.

Eunápolis, ____ de ______________ de 2023

Guardian's signature: ____

Researcher's signature: ______________________________________

Ricardo Toshio Fujiwara

Endereço: Universidade Federal de Minas Gerais - Instituto de Ciências Biológicas, bloco E, 4º andar, sala 173. Av. Pres. Antônio Carlos, 6627 - Pampulha, Belo Horizonte – MG

Universidade Federal do Sul da Bahia, Campus Paulo Freire - Praça Joana Angélica, 250, bairro São José, Teixeira de Freitas – BA. e-mail: ppgsab@ufsb.edu.br
